# 609 Illuminating the Impact of Social Vulnerability Index on Acute Burn Outcomes

**DOI:** 10.1093/jbcr/iraf019.238

**Published:** 2025-04-01

**Authors:** Alexis Henderson, Hilary Liu, José Arellano, Mare Kaulakis, Christopher Fedor, Garth Elias, Alain Corcos, Jenny Ziembicki, Francesco Egro

**Affiliations:** University of Pittsburgh School of Medicine; University of Pittsburgh Medical Center; University of Pittsburgh Medical Center; University of Pittsburgh School of Medicine; University of Pittsburgh School of Medicine; University of Pittsburgh Medical Center; University of Pittsburgh Medical Center; University of Pittsburgh Medical Center; University of Pittsburgh Medical Center

## Abstract

**Introduction:**

The social vulnerability index is a composite value scoring census tracts across the country from 0-1 on 16 US Census variables within 4 overarching themes: socioeconomic status, household composition and disability, minority status and language, and housing type and transportations. Scores closer to 0 indicate more vulnerable areas. This study aims to explore the impact of social vulnerability on acute burn outcomes.

**Methods:**

A retrospective review was conducted on patients who presented to a single ABA-verified burn center with acute burn injuries between 2016 and 2023. Data collection included demographics, distance to burn center, burn characteristics, and outcomes. Multiple logistic regression and analysis of variance were performed to analyze the effect of Social Vulnerability Index (SVI) and on burn outcomes.

**Results:**

A total of 2515 patients (mean age 41.2 ± 24.6 years; 82.2% Caucasian, 13.2% African American, 2.14% Native Hawaiian/Pacific Islander, 0.93% American Indian/Alaska Native) were included. The average SVI value was 0.384 ± 0.213, and time from injury to admission was 1.02 ± 2.9 days. Most of the patients (74.0%, n=1861) had 2nd or 3rd degree burns, with a mean TBSA of 7.4 ± 12.7%. Most of these burns were 2nd degree (93.0%; n= 1730). Caucasians had the lowest average SVI (0.38 ± 0.21), and Native Hawaiians/Pacific Islanders had the highest (0.45 ± 0.24).

SVI was not significantly associated with mortality (χ²=2.47; p=0.120), ventilator days (β=0.559; p=0.217), operations (β=-0.242; p=0.155), TBSA (β=0.195; p=0.892), hospital days (β=-1.098; p=0.608), ICU days (β=-0.764; p=0.445), longer delays between injury and admission (β=-0.309; p=0.385), or between racial groups (χ²=6.12; p= 0.190).

**Conclusions:**

SVI, a composite score of socioeconomic status, household composition and disability, minority status and language, and housing type and transportation, did not have a significant effect on burn outcomes.

**Applicability of Research to Practice:**

SVI in our practice was not necessarily associated with poor outcomes, however, confounding individual factors within the SVI should be further assessed and possibly taken into consideration among healthcare professionals treating underserved and vulnerable populations.

**Funding for the Study:**

N/A

